# Survey of bioinformatics courses and concentrations in ALA-accredited master’s programs

**DOI:** 10.29173/jchla29617

**Published:** 2022-08-04

**Authors:** Leah Graham, Melissa Moleirinho

**Affiliations:** MISt Student. McGill University, Montreal, QC, Canada.

## Abstract

**Introduction:**

The interdisciplinary field of bioinformatics is considered an information-based discipline by many. Yet, it is unclear how Master of Library and Information Science (MLIS) degrees prepare librarians to apply their expertise in this unique, often non-textual information environment. The goal of this study is to identify the availability of MLIS-based bioinformatics educational opportunities to provide an update to the current bioinformatics landscape in North American MLIS programs or iSchools.

**Methods:**

We conducted a survey of available bioinformatics courses and program concentrations within 69 ALA-accredited master’s programs. Using course catalogues and program descriptions on department websites, we identified the existence of courses and concentrations specific or related to the field of bioinformatics. We also surveyed the availability of associated certificate programs or degree alternatives.

**Results:**

Only two library and information science (LIS)-based bioinformatics courses are currently offered to MLIS students in ALA-accredited programs. There are no bioinformatics concentrations offered in the programs surveyed, however two graduate certificates could be applied towards an ALA-accredited master’s degree. Students interested in related fields can pursue degree alternatives, including eight dual degree options.

**Discussion:**

The scarcity of LIS-based bioinformatics courses and program concentrations may suggest that LIS has not adopted bioinformatics into their field nor curricula. As a result, students interested in pursuing careers in bioinformatics and related disciplines must actively seek out opportunities for education and professional development. Bioinformatics degree options within MLIS or iSchools points towards an increased dialogue and acceptance of the connection between bioinformatics and information science, but the lack of ALA -accreditation limits possibilities for emerging librarians.

## Introduction

With the completion of the Human Genome Project in 2003, the field of bioinformatics was thrust into a new age of research possibilities, innovation, and resources. The availability of molecular biology database resources has continued to increase, as illustrated by the *Nucleic Acids Research* database issues. A total of 548 databases were compiled in 2004 [1], in contrast to the most recent issue citing a total of 1,641 curated databases [2]. Bioinformatics is an interdisciplinary field, intersecting the biological sciences, computer science, and library and information science (LIS), however its place within any of these fields has been long debated. Yet, many in the LIS field view bioinformatics as an information-based discipline [3-7]. Despite the increase in prominence and continued development of bioinformatics, it is still unclear how the LIS field trains librarians to apply their own professional expertise in this unique and often non-textual information environment.

Citing the “importance of applying principles of knowledge organization and information retrieval (e.g., classification, metadata, vocabulary control, etc.) in the effective handling of the huge amounts of biological information generated in the laboratory” [6], the appearance of information professionals in the field is not surprising. With titles like “Bioinformatics Librarian” appearing in health sciences libraries of the early 2000s [8], discussions arose around the LIS-based education and related experience required of information professionals in bioinformatics, as well as how it should be obtained [5-7,9,10,11,12]. However, it is unclear how this emerging field has influenced the development of Master of Library and Information Science (MLIS) curricula pertaining to bioinformatics.

Understandings of the fundamentals of bioinformatics instruction from an LIS-perspective vary across the board. Bartlett (2005) noted the importance of teaching library science skills, such as providing reference services and formal resource instruction of bioinformatics resources, as well as the LIS theory and ethical considerations inherent to bioinformatics [3]. However, other approaches additionally considered the importance of knowledge management (e.g., research data management), writing [13], and importantly the contextual knowledge and/or practical experience in the field [8,11,14].

Based on existing research of the landscape, LIS-based bioinformatics training has been limited in availability. For instance, in 2004 Helms et al. identified the University of North Carolina (UNC) at Chapel Hill’s Specialization in Bioinformatics certificate awarded with either the Master of Library Science (MLS) or Master of Information Science (MIS) degree as the only bioinformatics training embedded in formally recognized LIS education [13]. Described as the first of its kind in Canada, McGill University’s Graduate School of Library and Information Studies (now School of Information Studies) first offered a LIS-based course in bioinformatics in 2005 that focused on library-based resources and services [3]. The only other master’s level bioinformatics degree identified in literature is that offered by the University of Illinois’ Graduate School of Library and Information, a joint endeavour with the Illinois Informatics Institute obtained by completing a combination of LIS, computer science, and biological science courses [9]. Given the interdisciplinary and cross-disciplinary nature of bioinformatics, courses on the topic vary significantly and may be found across the university [5,9].

Most librarians wishing to work professionally in the bioinformatics field would be required to obtain training additional to their MLIS degree [11,13], such as continuing education in the biological and health sciences, professional experience in a laboratory, or workshops offered through the Medical Library Association and the National Center for Biotechnology Information [8]. This seems to represent a larger trend in LIS-based health sciences instruction through inter-departmental or inter-faculty collaboration, as demonstrated by the University of Southern Mississippi’s School of Information Science’s collaboration with the Department of Public Health [7] and the University of North Texas’ Department of Library Science 5-week course “Genomics and Translational Medicine for Information Professionals” taught by a bioinformaticist [15]. As a result, the importance of interdisciplinary dialogue as the field of LIS and the need to organize, store, and retrieve information permeates all others [10].

Several concerns related to the development of LIS bioinformatics instruction have been raised, including the need to define the scope of bioinformatics for LIS purposes, to identify opportunities for collaborations and partnerships with bioinformatics centres and institutes, and to clarify the required level of background education in sciences [9]. While, overall, there is a clear sense that there is a need for bioinformatics professionals with LIS foundations [4-5,9-11], the sparse and disparate literature of the 2010s place the emphasis and inquiry more broadly on LIS-preparation for fields like eScience [12] and library-based collaboration for health informatics instruction [7,14], and provide little evidence of the current state of bioinformatics education in the field of LIS.

In this study, the 64 current LIS schools offering programs accredited by the American Library Association (ALA) were reviewed to present an up-to-date picture of the state of LIS-based bioinformatics instruction in North America. The work was guided by the following questions:

- How many ALA-accredited master’s programs offer concentrations in bioinformatics?
- How many bioinformatics courses are offered as part of the ALA-accredited master’s programs?
- What bioinformatics degree programs are offered by LIS schools in collaboration with other departments or organisations? Are these programs ALA-accredited?

## Methods

### Framework for data collection and analysis

In this study, concentrations refer to specific courses of study or pathways within an ALA-accredited master’s degree. Certificate programs offered by LIS or iSchools refer to additional qualifications for professional development or subject-specific certificates applied to a master’s degree. Joint degree programs offered by these schools that were either specific or related to bioinformatics were also noted. With regards to the professional practice of LIS-trained librarians and information professionals, those working in related fields will also need basic knowledge of this non-textual data [16]. As a result, educational opportunities that relate to the field of bioinformatics, despite not directly referencing it, are relevant to our context. While students enrolled in LIS programs often have the opportunity to enroll in courses outside their department (with or without administrative approval) for elective credits or in completion of a dual degree, this study is concerned solely with bioinformatics courses offered by LIS-specific departments.

In preparation for the data collection process, we defined two categories according to the level of relation to bioinformatics *: (i)* bioinformatics-specific; *and (ii)* bioinformatics-related. The bioinformatics-specific category encompasses program concentrations/courses that are focused on or make specific mention of bioinformatics. In comparison, the bioinformatics-related category is characterized by concentrations, courses, or programs that tie into the broader categories of health sciences information or informatics and science and technology.

The second category of bioinformatics-related program concentrations or courses was included to better inform and contextualize the availability of bioinformatics training identified. As the ALA identify concentrations and career pathway options in their directory such as “Health Sciences Librarianship/Health Informatics” and “Science Librarianship”, we considered the possibility that bioinformatics training could be embedded within broader courses. The course or program titles and descriptions included in this category do not directly reference bioinformatics, however they reflect the possible points of entry that information professionals in-training may have into the field of bioinformatics.

### Data collection

As this study focuses on the availability of bioinformatics education and training to students pursuing an ALA-accredited master’s degree, we used the ALA’s directory of schools (current as of 18 March 2021) as a starting point. To identify the availability of bioinformatics-specific concentrations or courses within these schools, we used Google’s advanced search function to search for each school and program webpages that could include this type of information, including program guides, course catalogues, curriculum summaries, etc. Essentially, search strategies consisted of combining the university or department or program name(s) and “course catalog”. Once on a department or program page, if necessary, we navigated to related pages using the webpages’ internal menus.

The collection of program concentrations and courses according to the bioinformatics-specific and bioinformatics-related categories was completed by both browsing and keyword searching the appropriate program and course descriptions. These searches were performed in March and April 2021 and information was drawn from the most up-to-date source available at the time of collection. Once located, either the course catalogue’s embedded search engines or the standard OS search function (i.e., Control-F) were used to search the following keywords for bioinformatics-specific courses: “bioinformatics”, “genomics”, and “genetics”. For bioinformatics-related courses, search terms such as “health”, “medical”, “biology”, and “science” (i.e., biological sciences) were searched to determine inclusion. In addition to the keyword searching, careful browsing of full course lists was used to identify relevant courses. Any associated graduate certificates and degree options available on LIS or iSchool websites were also collected.

## Data Analysis

A total of 69 ALA-accredited MLIS programs at 64 LIS or iSchools were identified for analysis. The data collected was compiled in a Microsoft Excel spreadsheet under categories noting the available bioinformatics-specific or related program concentrations, certificates, degree alternatives, and courses. Employing descriptive statistical analysis, the data was analyzed primarily by frequency to demonstrate the total occurrence of opportunities available in a MLIS degree in each category.

## Results

The results are divided and organized into three distinct categories: courses, program concentrations, and degree alternatives. The complete results of this survey are available in Online Supplement, Appendix A.

### Courses

**Fig. 1:**
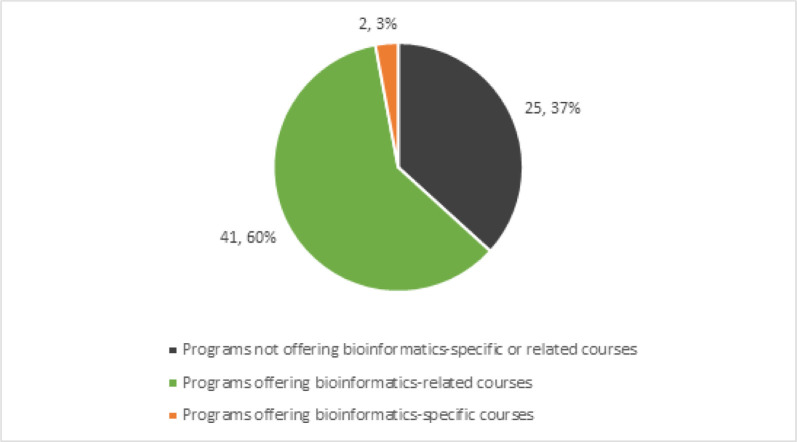
Programs offering bioinformatics-specific or related courses

Of the 68 ALA-accredited master’s programs for which the course catalogue was available, only Drexel University’s College of Computing and Informatics and McGill University’s School of Information Studies offer the bioinformatics-specific courses INFO648: Healthcare Informatics and GLIS673: Bioinformatics Resources, respectively. Of a total of 68 ALA-accredited LIS graduate programs, only 2 (3%) have bioinformatics-specific courses (Fig. 1).

43 programs (63%) surveyed offer at least one course related to bioinformatics (e.g., Health informatics or Health sciences, Medical librarianship, Resources for science and technology, etc.), including the two aforementioned programs offering bioinformatics-specific courses. In several cases, course descriptions provide too little information related to the course content. As such, it is possible that there are more courses, such as some listed as bioinformatics-related courses, that include bioinformatics instruction and training for LIS students.

### Program concentrations

None of the 69 programs surveyed offer integrated bioinformatics-specific concentrations, while 6 programs (9%) offer bioinformatics-related concentrations (Table 1). These concentrations are meant to guide students with specific interests in their course selection and pathway through the degree program.

**Table 1:** Bioinformatics-related concentrations offered as part of ALA-accredited degrees

University	Degree	Concentration
Emporia State University	Master of Library Science	Health Information Professionals
Florida State University	Master of Science in Information	Health Informatics
Texas Woman’s University	Master of Arts in Library Science	Health Science Libraries Program Track
Texas Woman’s University	Master of Library Science	Health Science Libraries Program Track
University of Arizona	Master of Arts in Library and Information Science	Health Sciences Librarianship/Health Informatics
University of Tennessee	Master of Science in Information Science	Science Information Pathway

Thirteen programs (19%) offer graduate-level certificates specific or related to bioinformatics. These certificates may be awarded independently or in concert with a graduate degree. Only two programs (3%) offer a bioinformatics-specific certificate which may be obtained as part of an ALA-accredited degree (Table 2). For example, UNC Chapel Hill offers the graduate certificate in bioinformatics in conjunction with the MSLS or MSIS degree; obtaining this certificate is dependent on additional requirements, including the completion of bioinformatics-related coursework and a significant project in the area.

**Table 2:** Bioinformatics-specific Certificate Programs Available with ALA-accredited degrees

University	Certificate	Required Conditions
University of Iowa	Graduate Certificate in Informatics (option to specialize in Bioinformatics)	May be awarded independently; may be applied towards aMaster of Arts in Library and Information Science
University of North Carolina Chapel Hill	Graduate Certificates in Bioinformatics, Biomedical Imaging Science, Public Health Informatics, Clinical Information Science	Must be awarded with a Master of Science in Library Science or Master of Science in Information Science

### Degree Alternatives

Of the 64 schools surveyed, there are a total of 11 degree alternatives (17%) available in partnership with or within LIS or iSchools for students interested in fields related to bioinformatics. As previously operationalized, in this context, bioinformatics-related is a category including fields like health informatics, health information, public health, and science (i.e., biology). Of these 11 degree alternatives, there are 8 dual degree options which permit students to obtain an ALA-accredited degree in conjunction with another bioinformatics-related degree (Table 3).

**Table 3:** Dual degree options: ALA-accredited degree & bioinformatics-related degree

University	Dual Degree Option
Kent State University	MLIS / MS in Health Informatics
Long Island University	MLS / MA or MS (planned by student)
Louisiana State University	MLIS / MS (planned by student, e.g., MS Biomedical sciences -Bioinformatics track)
Texas Woman’s University	MLS / MS Health Studies
Texas Woman’s University	MA in Library Science / MS Health Studies
Catholic University of America	MSLIS / MS Biology
University of North Carolina at Chapel Hill	MSIS / Master of Healthcare Administration or MS of Public Health
University of Wisconsin-Milwaukee	MLIS / MS Health Care Informatics

There are three other degree options that are LIS-affiliated, meaning they are offered by the LIS or iSchool, often in collaboration with other university departments (Table 4). Despite this tie to the LIS field, these degree alternatives are not ALA-accredited. The only bioinformatics-specific degree option is offered by the University of Illinois at Urbana-Champaign's School of Information Science.

**Table 4:** LIS-affiliated bioinformatics-specific or related degree alternatives (not ALA-accredited)

University	Alternative Degree Option
University of Illinois at Urbana-Champaign	MS in Bioinformatics –Information Science Concentration (in collaboration with the Dept. of Animal Sciences, Dept. of Computer Science, Dept. of Crop Sciences)
University of Michigan	Master of Health Informatics (in collaboration with the School of Public Health and the Medical School)
Western University	Master of Health Information Science (in collaboration with the Faculty of Health Sciences)

## Discussion

Results showed only two LIS-based bioinformatics course offerings for MLIS students enrolled in ALA-accredited programs. There were no program concentrations offered, but two graduate certificate options can be applied towards an ALA-accredited master’s degree. Eight dual degree options are offered to MLIS students in fields related to bioinformatics, while three iSchools offer non-ALA-accredited master’s programs. The findings demonstrate that there are few opportunities for current LIS students to explore and specialize in bioinformatics during ALA-accredited MLIS degrees. By distancing the study and practice of bioinformatics from accredited library education programs, the role of the librarian and information professional in bioinformatics is put into question.

### Few bioinformatics education opportunities for current LIS students

The scarcity of LIS-based bioinformatics courses and program concentrations may suggest that LIS has not adopted bioinformatics into their field nor curricula. This conflicts with the expectations found in the early 2000s literature, which predicted the increased relevance of the librarian or information professional in this field. In our survey, McGill University’s course GLIS673 Bioinformatics Resources is the only course dedicated to library training in bioinformatics resources for current MLIS students. As described in 2005 and still presently, it emphasizes the LIS perspective in the field of bioinformatics, including recognizing the information behaviours and needs of patrons, as well as search and retrieval of both literature and non-textual information (e.g., protein sequences) [17].

Drexel University’s equivalent, INFO648: Healthcare Informatics, is broader in its curriculum yet describes an aspect of the course which focuses on library training in bioinformatics education and research contexts. It is the only other course available to MLIS students which explicitly states the instruction of bioinformatics for these contexts, likely available as it is a cross-listed course with the other department of information science programs, such as the Master of Science in Health Informatics [18]. Additionally, the course bears similarity, by name alone, to various bioinformatics-related courses, as well as program concentrations, identified commonly in the fields of health and healthcare informatics. While it is unclear whether these courses and program concentrations include bioinformatics components in their curriculum, it is clearly not advertised as a focal point. As LIS-based health, medical, and sciences courses may be an entry point for those interested in bioinformatics, interest may be impacted by the lack of courses and concentrations available.

Due to this limitation, students interested in pursuing careers in bioinformatics and related disciplines must actively seek out opportunities for education and professional development. Integrated bioinformatics-specific certificate programs provide opportunities for students to specialize while completing their degree; however, this type of option is limited to only two schools (University of Iowa and UNC Chapel Hill, see Table 2). Independent certificate programs offered by LIS or iSchools enable students or professionals to update their qualifications after the completion of their ALA-accredited degree. Petrinic and Urquhart (2007) found that continuing professional development workshops and certificate programs were required for health sciences librarians in order to meet the developing professional requirements of their employment contexts [19]. By opting for integrated or additional certification within a LIS department or school, students and information professionals are more likely to obtain bioinformatics training that is reflective of and pertinent to a LIS context. While not uncommon for LIS professionals (e.g., academic subject area or liaison librarians) to seek out additional qualifications in their field of interest, the lack of program concentrations may put into question the role of librarians in the field of bioinformatics.

### Role of LIS professionals in bioinformatics

The prominence of alternate degree routes, in particular the dual-degree options available to LIS students, reflects the interdisciplinary nature of the field and the general calls for collaboration noted in the literature. While an interested LIS student would likely take a primarily information science perspective to bioinformatics, a strong grasp of the biological and laboratory context is crucial [8, 11, 14]. In many ways, the clear availability of the health informatics concentration and dual degree options suggests that informationists, typically professionals in the fields of healthcare or biological sciences with the skillset of information retrieval [20], may be taking up this role in bioinformatics contexts. Dalrymple et al. (2011) noted that this role has evolved “from a librarian who possesses additional domain knowledge but remains aligned with librarianship, to an information professional whose educational preparation is intentionally aimed at healthcare” [10]. This suggests their expertise in the field will contribute to their ability to effectively provide library services [15].

However, the appearance of bioinformatics-specific and related degree options within LIS or iSchools points towards an increased dialogue and acceptance of the connection between bioinformatics and information science as information-based disciplines. For instance, the University of Illinois at Urbana-Champaign's School of Information Sciences offers the MS in Bioinformatics — one of four campus units to do so. The information page for this degree explicitly states that there is a “critical demand” for these kinds of professionals [21]. However, the degree is not ALA-accredited. Keeping in mind that many employers and states require it, the lack of ALA-accreditation may limit the capacity for these trained information professionals to bridge the gap and enter librarianship. As a result, qualified graduates with expertise may be unable to enter a context that needs them; on the flip side, students interested in bioinformatics may forego this degree in favour of a general ALA-accredited degree, due to the employment flexibility it provides.

The librarian’s role in bioinformatics service provision recognizes the value of their expertise in this specific information context. Yarfitz and Ketchell’s (2000) article noted the success of centralizing bioinformatics services, including consultation, education and training, and resource development, within the library [22]. There is a demonstrated need for library services that can meet the bioinformatics informational needs of researchers and students through specialized instruction and support [23]. Geer (2006) argues that the library is a natural fit for this type of service provision [24]. Despite their unique capacity to meet these critical needs, as it stands, our findings show that LIS or iSchools have yet to concentrate efforts into creating emerging librarians to meet them. Lyon (2008) notes “these specialists are not easy to locate and hire” [25] and librarians often rely on bioinformatics specialists to assist with technical and field-based skills [14]. For institutions facing a need for medical and science librarians with the relevant training, this is a matter of urgency that ALA-accredited degree programs are not poised to resolve.

### Limitations

This research endeavour is not without limitations. By design, this study only accounts for LIS graduate programs with ALA-accreditation (and those affiliated with schools housing ALA-accredited programs, for discussion), which limits our focus to a North American context. In the United States and Canada, an ALA-accredited degree is often a requirement for librarian positions; this limitation is reflective of our desire to consider the implications of bioinformatics education or training for students entering this specific professional context. This study was also limited by the availability of course information online, as the availability and level of detail for descriptions of course offerings differed from institution to institution.

### Future research

Further research could use similar methods to expand out of the North American context by surveying the roughly 120 LIS schools within the iSchools organization globally. As this study relied on the availability of course information online, a future study could reach out to each program directly to better understand when courses are offered, what topics are covered, as well as attendance rates (both frequency and demographics). Finally, as it is clear from these findings that the field of LIS has not embraced bioinformatics on the whole, future research should consider LIS student experiences in the available collaborative degrees and related non-LIS courses. Are students opting for these alternatives obtaining what they seek – LIS-relevant bioinformatics education? Leaving the ALA-accredited program context, future research could additionally consider other avenues for bioinformatics education available to librarians and what skills and perspectives are being prioritized in this training.

## Conclusion

Despite expectations of bioinformatics alignment with information science noted in the literature, it appears that the ALA have yet to offer a clear pipeline for emerging librarians to gain the required knowledge of genetics and molecular biology resources. As a result, while the literature describes a demand for LIS professionals in a variety of health sciences settings, including bioinformatics units, it seems unlikely that these professionals would come from a LIS foundation.

However, the unique expertise of librarians and information professionals lends itself to proficiency in the field of bioinformatics. ALA-accredited schools endeavouring to create new generations of in-demand professionals can harness this opportunity to nurture a specific skill set in interested students, whether by offering new courses or specializations, or partnering with related departments for the creation of new accredited programs or degree alternatives. As we look to the future, growing awareness of how librarians can contribute to this interdisciplinary field could result in increased integration of bioinformatics education. Time will tell whether programs will evolve to create librarians who graduate with the expertise to meet the demand or whether librarians themselves will have to bear the brunt of developing these skills postgraduation.

## Supplementary Material


