# Supplementary material for: Survey of bioinformatics courses and concentrations in ALA-accredited master’s programs
Source: J Can Health Libr Assoc. 2022 Aug 4;43(2):58–67. doi: 10.29173/jchla29617 (PMC9359087; doi:10.29173/jchla29617)
Supplement: Supplementary file 1 [file JCHLA-43-058-s001.pdf]

Appendix A

To improve the display, table headers are abbreviated. See below for the full table headings:

Uni = University and location

Prog = ALA-accredited degree program name

Bio-S/R Cert(s) = Bioinformatics-specific/related certificate(s)

Bio-S/R Deg Alts = Bioinformatics-specific/related degree alternatives

Bio-S/R Conc(s) = Bioinformatics-specific/related concentration(s)

Bio-S Course(s) = Bioinformatics-specific course(s)

Bio-R Course(s) = Bioinformatics-related course(s)

Uni	Prog	Bio-S/R Cert(s)	Bio-S/R Deg Alts	Bio-S/R Conc(s)	Bio-S Course(s)	Bio-R Course(s)
Chicago State University<br><br>Chicago, IL, US	Master of Science in Library and Information Science	No	No	No	No	• LSC 870: Health Sciences Information<br>• LSC 871: Health Informatics

Clarion University of Pennsylvania<br><br>Clarion, PA, US	Master of Science in Library Science	No	No	No	No	• LS 592: Consumer Health Informatics<br>• LS 593: Health Science Librarianship
Dalhousie University<br><br>Halifax, NS, CA	Master of Information	No	No	No	No	• INFO 6750.03: Health Science Literature and Information Sources
Dominican University<br><br>River Forest, IL, US	Master of Library and Information Science	No	No	No	No	• LIS 794: Foundations of Health Informatics
Drexel University	Master of Science in Information -	Graduate Minor in Healthcare Informatics	MS in Health Informatics (not ALA-accredited)	No	INFO 648: Healthcare Informatics	• INFO 731: Managing Health Informatics Project

Philadelphia, PA, US	Library & Information Science					• INFO 732: Healthcare Informatics: Planning & Evaluation<br>• INFO 733: Public Health Informatics
East Carolina University<br><br>Greenville, NC, US	Master of Library Science	No	No	No	No	No
Emporia State University<br><br>Emporia, KS, US	Master of Library Science	Health Information Professionals Certificate	No	Library science, health information professionals concentration	No	• LI 881: Health Science Librarianship<br>• LI 886: Consumer Health Information
Florida State University	Master of Science in Information	Graduate Certificate in Health Informatics	No	Health Informatics	No	• LIS 5418: Introduction to Health Informatics

Tallahassee, FL, US						• LIS 5419: Consumer Health Informatics<br>• LIS 5631: Health Information Sources<br>• LIS 5788: Management of Health Information Technology
Florida State University<br><br>Tallahassee, FL, US	Master of Arts in Information	Graduate Certificate in Health Informatics	No	No	No	• LIS 5418: Introduction to Health Informatics<br>• LIS 5419: Consumer Health Informatics<br>• LIS 5631: Health Information Sources<br>• LIS 5788: Management of Health Information Technology

Indiana University (Purdue University Indianapolis)<br><br>Indianapolis, IN, US	Master of Library and Information Science	No	No	No	No	• LIS-S 653: Health Sciences Librarianship
Indiana University Bloomington<br><br>Bloomington, IN, US	Master of Library Science	No	No	No	No	No
Indiana University Bloomington	Master of Information Science	No	No	No	No	No

Bloomington, IN, US						
Kent State University<br><br>Kent, OH, US	Master of Library and Information Science	Post-Baccalaureate Certificate in Health Informatics	Dual Degrees: MLIS / MS in Health Informatics	No	No	• LIS 60622 (80622): Science/Technology Information Sources and Services<br>• LIS 60620 (80620): Health Information Resources
Long Island University<br><br>Brookville, NY, US	Master of Science in Library and Information Science	No	Dual Degrees: MLS / MA or MS (planned by student)	No	No	• LIS 916: Health Sciences Libraries
Louisiana State University	Master of Library and Information Science		Dual Degrees: MLIS / Master of Science (planned by student)	No	No	• LIS 7202: Resources for Science and Technology

Baton Rouge, LA, US						
McGill University<br><br>Montreal, QC, CA	Master of Information Studies	No	No	No	GLIS 673: Bioinformatics Resources	• GLIS 671: Health Sciences Information
North Carolina Central University<br><br>Durham, NC, US	Master of Library Science	No	No	No	No	• LSIS 5015: Introduction to Health Informatics<br>• LSIS 5245: Health Sciences Resources and Services
Pratt Institute<br><br>New York, NY, US	Master of Science in Library and Information Science	No	No	No	No	No

Queens College (CUNY)<br><br>Flushing, NY, US	Master of Library Science	No	No	No	No	No
Rutgers, The State University of New Jersey<br><br>New Brunswick, NJ, US	Master of Information	No	No	No	No	• 17:610:545: Health Sciences Information
San Jose State University<br><br>San Jose, CA, US	Master of Library and Information Science	No	No	No	No	No

Simmons University<br><br>Boston, MA, US	MS Library and Information Science	No	No	No	No	No
Southern Connecticut State University<br><br>New Haven, CT, US	Master of Library and Information Science	No	No	No	No	No
St. Catherine University<br><br>St. Paul, MN, US	Master of Library and Information Science	No	No	No	No	No

St. John's University<br><br>Queens, NY, US	Master of Science in Library and Information Science	No	No	No	No	No
Syracuse University<br><br>Syracuse, NY, US	Master of Science in Library and Information Science	No	No	No	No	No
Texas Woman's University<br><br>Denton, TX, US	Master of Library Science	Post-Master's certificate in Evidence-Based Health Science Librarianship	Dual Degrees: MLS / MS Health Studies	Health Science Libraries Program track	No	• LS 5363: Health Sciences Information Services Management<br>• LS 5473: Health Reference Services and Resources

						• LS 5483: Patient and Consumer Health Information
Texas Woman's University<br><br>Denton, TX, US	Master of Arts in library Science	Post-Master's certificate in Evidence-Based Health Science Librarianship	Dual Degrees: MA in Library Science / MS Health Studies	Health Science Libraries Program track	No	• LS 5363: Health Sciences Information Services Management<br>• LS 5473: Health Reference Services and Resources<br>• LS 5483: Patient and Consumer Health Information
The Catholic University of America<br><br>Washington, DC, US	Master of Library and Information science	No	Dual Degrees: MSLIS / MS Biology	No	No	No

The University of Southern Mississippi<br><br>Hattiesburg, MS, US	Master of Library and Information Science	No	No	No	No	• LIS 667: Health Informatics
Université de Montréal<br><br>Montreal, QC, CA	Maitrise en sciences de l'information	No	No	No	No	No
University at Albany (SUNY)<br><br>Albany, NY, US	Master of Science in Information Science	No	No	No	No	No

University at Buffalo (SUNY)<br><br>Buffalo, NY, US	Master of Science in Information and Library Science	No	No	No	No	No
University at Buffalo (SUNY)<br><br>Buffalo, NY, US	Master of Science in School Librarianship	No	No	No	No	No
University of Alabama<br><br>Tuscaloosa, AL, US	Master of Library and Information Studies	No	No	No	No	No

University of Alberta<br><br>Edmonton, AB, CA	Master of Library and Information Studies	No	No	No	No	• LIS 520: Information Resources in Specialized fields
University of Arizona<br><br>Tucson, AZ, US	Master of Arts in Library and Information Science	Graduate Certificate in Medical and Community Health Information	No	Health Sciences Librarianship/Health Informatics	No	• LIS 646: Healthcare informatics: Theory and practice<br>• LIS 624: Community Health and Medical Informatics<br>• LIS 533: Medical Online Searching<br>• LIS 556: Health Information in Ethnic-Cultural Communities
University of British Columbia	Master of Library and	No	No	No	No	• LIBR 534: Health Information Sources and Services

Vancouver, BC, CA	Information Studies					
University of California, Los Angeles<br><br>Los Angeles, CA, US	Master of Library and Information Science	No	No	No	No	• M 253: Medical Knowledge Representation<br>• M 254: Medical Information Infrastructures and Internet Technologies<br>• M 255: Medical Decision-making
University of Denver<br><br>Denver, CO, US	Master of Library and Information Science	No	No	No	No	No
University of Hawaii	Master of Library and	No	No	No	No	No

Honolulu, HI, US	Information Science					
University of Illinois at Urbana-Champaign<br><br>Champaign, IL, US	Master of Science in Library and Information Science	No	iSchool offers: MS in Bioinformatics IS concentration (in collaboration with the Dept. of Animal Sciences, Dept. of Computer Science, Dept. of Crop Sciences) (not ALA-accredited)	No	No	• IS 591 HIS: Health Science Information Services & Resources<br>• IS 597 PD: Practical Health Data Analytics<br>• IS 596 GH: Global Health Informatics
University of Iowa<br><br>Iowa City, IA, US	Master of Arts in Library and Information Science	Graduate Certificate in Informatics (Bioinformatics option)	No	No	No	• SLIS 5900: Health Informatics<br>• SLIS 5950: Health Information and Communication

University of Kentucky<br><br>Lexington, KY, US	Master of Science in Library Science	No	No	No	No	• LIS 626: Electronic Information Resources in the Health Sciences<br>• LIS 627: Consumer Health Information Resources<br>• LIS 629: Introduction to Medical Informatics<br>• LIS 640: Health Information Resource Services
University of Maryland<br><br>College Park, MD, US	Master of Library and Information Science	No	No	No	No	• INFM 728K: Consumer Health Informatics<br>• INST 680: Health Informatics

						• INST 681: Health Information Behaviour<br>• INST 682: Personal Health Informatics and Visualization
University of Michigan<br><br>Ann Arbor, MI, US	Master of Science in Information	Graduate Certificate in Health Informatics	School of Information offers: Master of Health Informatics (in collaboration with the School of Public Health and the Medical School) (not ALA-accredited)	No	No	• 542: Introduction to Health Informatics<br>• 554: Consumer Health Informatics<br>• 574: Health Informatics Program Seminar<br>• 611: Population Health Informatics<br>• 661: Managing Health Informatics<br>• 648: Evaluation Methods in Health

						Informatics and Learning Systems<br>• 654: Critical Policy Issues in Health IT<br>• 684: Designing Consumer-Health Technologies
University of Missouri<br><br>Columbia, MO, US	Master of Library and Information Science	No	No	No	No	• 9415: Fundamentals of Medical Librianship
University of North Carolina at Chapel Hill<br><br>Chapel Hill, NC, US	Master of Science in Library Science	Graduate Certificates in Bioinformatics, Biomedical Imaging Science, Public Health Informatics,	No	No	No	• INLS 515: Consumer Health Information<br>• INLS 706: Biomedical Informatics Research Review

		Clinical Information Science				• INLS 770: Health Informatics Seminar<br>• INLS 793: Health Informatics Practicum<br>• INLS 748: Health Sciences Environment<br>• INLS 705: Health Sciences Information<br>• INLS 725: Electronic Health Records<br>• INLS 710: Evidence-based medicine
University of North Carolina at Chapel Hill<br><br>Chapel Hill, NC, US	Master of Science in Information Science	Graduate Certificates in Bioinformatics, Biomedical Imaging Science, Public Health Informatics,	Dual Degrees: MSIS / Master of Healthcare Administration or Masters of Science of Public Health	No	No	• INLS 515: Consumer Health Information<br>• INLS 706: Biomedical Informatics Research Review

		Clinical Information Science				• INLS 770: Health Informatics Seminar<br>• INLS 793: Health Informatics Practicum<br>• INLS 748: Health Sciences Environment<br>• INLS 705: Health Sciences Information<br>• INLS 725: Electronic Health Records<br>• INLS 710: Evidence-based medicine
University of North Carolina at Greensboro<br><br>Greensboro, NC, US	Master of Library and Information Studies			No	No	No

University of North Texas<br><br>Denton, TX, US	Master of Science - Library Science	No	No	No	No	• INFO 5365: Health Sciences Information Management<br>• INFO 5636: Community-based health Information<br>• INFO 5637: Medical Informatics
University of Oklahoma<br><br>Norman, OK, US	Master of Library and Information Studies	No	No	No	No	• LIS 5133: Biomedical Bibliography and Reference Materials<br>• LIS 5163: Biomedical Databases<br>• LIS 5827: Internship (in a medical or health information setting)<br>• LIS 5970: Health Informatics

						• LIS 5970: Community Health Information
University of Ottawa<br><br>Ottawa, ON, CA	Master of Information Studies	No	No	No	No	No
University of Pittsburgh<br><br>Pittsburgh, PA, US	Master of Library and Information Science	No	No	No	No	• LIS 2587: Applications in Medical Informatics<br>• LIS 2585: Health Consumer Resources and Services<br>• LIS 2586: Health Sciences Resources and Services

University of Puerto Rico<br><br>San Juan, PR (US*)	Master of Information Sciences	No	No	No	Course catalogue was not located	Course catalogue was not located
University of Rhode Island<br><br>Kingston, RI, US	Master of Library and Information Studies	No	No	No	No	• LSC 537: Health Sciences Librarianship<br>• LSC 542: Library Materials in Science and Technology
University of South Carolina<br><br>Columbia, SC, US	Master of Library and Information Science	No	No	No	No	No
University of South Florida	Master of Arts in Library and	No	No	No	No	No

Tampa, FL, US	Information Science					
University of Southern California<br><br>Los Angeles, CA, US	Master of Management in Library and Information Science	No	No	No	No	• LIM 556: Health Sciences Libriansh ip
University of Tennessee<br><br>Knoxville, TN, US	Master of Science in Information Sciences	Graduate certificate in Health Informatics	No	Science Information Pathway	No	• 547: Health Sciences Information Centers
University of Texas at Austin	Master of Science in Information Studies	No	No	No	No	• INF 385V: Health Informatics<br>• INF385N: Informatics:

Austin, TX, US						Consumer Health Informatics
University of Toronto<br><br>Toronto, ON, CA	Master of Information	No	No	No	No	• INF 2135H: Evidence Based Healthcare for Librarians
University of Washington<br><br>Seattle, WA, US	Master of Library and Information Science	No	No	No	No	• LIS 528: Health Sciences Information Needs, Resources, and Environment
Western University<br><br>London, ON, CA	Master of Library and Information Science	No	Master of Health Information Science (in collaboration with Faculty of Health Sciences) (not ALA-accredited)	No	No	• FIMS 9325: Introduction to Health Informatics

University of Wisconsin-Madison<br><br>Madison, WI, US	Master of Arts in Library and Information Studies	No	No	No	No	• LIS 517: Digital Health: Information and Technologies supporting Consumers and Patients
University of Wisconsin-Milwaukee<br><br>Milwaukee, WI, US	Master of Library and Information Science	No	Coordinated Degree: MLIS & MS Health Care Informatics	No	No	• INFOST 835: Information Sources and Services in the Health Sciences
Valdosta State University<br><br>Valdosta, GA, US	Master of Library and Information Science	No	No	No	No	No

Wayne State University<br><br>Detroit, MI, US	Master of Library and Information Science	No	No	No	No	• 7610: Health Sciences Information Services and Resources<br>• 7620: Health Informatics
